# Role of microglia in a mouse model of paediatric traumatic brain injury

**DOI:** 10.1016/j.bbi.2016.11.001

**Published:** 2017-07

**Authors:** Vibol Chhor, Raffaella Moretti, Tifenn Le Charpentier, Stephanie Sigaut, Sophie Lebon, Leslie Schwendimann, Marie-Virginie Oré, Chiara Zuiani, Valentina Milan, Julien Josserand, Regina Vontell, Julien Pansiot, Vincent Degos, Chrysanthy Ikonomidou, Luigi Titomanlio, Henrik Hagberg, Pierre Gressens, Bobbi Fleiss

**Affiliations:** a PROTECT, INSERM, Unversité Paris Diderot, Sorbonne Paris Cité, Paris, France; b PremUP, Paris, France; c Department of Anesthesia and Intensive Care, Georges Pompidou European Hospital, Paris, France; d Università degli Studi di Udine, Udine, Italy; e Department of Perinatal Imaging and Health, Department of Division of Imaging Sciences and Biomedical Engineering, King’s College London, King’s Health Partners, St. Thomas’ Hospital, London SE1 7EH, United Kingdom; f Department of Anesthesia and Intensive Care, Pitié Salpétrière Hospital, F-75013 Paris, France; g Department of Neurology, University of Wisconsin, Madison, WI, USA; h Department of Clinical Sciences, Sahlgrenska Academy/East Hospital, Gothenburg University, 416 85 Gothenburg, Sweden

**Keywords:** Phenotype, Cytokine, Chemokine, Apoptosis, Neuron, Immature, Cerebral, Macrophage, Inflammation, Minocycline

## Abstract

The cognitive and behavioural deficits caused by traumatic brain injury (TBI) to the immature brain are more severe and persistent than TBI in the mature brain. Understanding this developmental sensitivity is critical as children under four years of age sustain TBI more frequently than any other age group. Microglia (MG), resident immune cells of the brain that mediate neuroinflammation, are activated following TBI in the immature brain. However, the type and temporal profile of this activation and the consequences of altering it are still largely unknown.

In a mouse model of closed head weight drop paediatric brain trauma, we characterized i) the temporal course of total cortical neuroinflammation and the phenotype of *ex vivo* isolated CD11B-positive microglia/macrophage (MG/MΦ) using a battery of 32 markers, and ii) neuropathological outcome 1 and 5 days post-injury. We also assessed the effects of targeting MG/MΦ activation directly, using minocycline a prototypical microglial activation antagonist, on these processes and outcome.

TBI induced a moderate increase in both pro- and anti-inflammatory cytokines/chemokines in the ipsilateral hemisphere. Isolated cortical MG/MΦ expressed increased levels of markers of endogenous reparatory/regenerative and immunomodulatory phenotypes compared with shams. Blocking MG/MΦ activation with minocycline at the time of injury and 1 and 2 days post-injury had only transient protective effects, reducing ventricular dilatation and cell death 1 day post-injury but having no effect on injury severity at 5 days.

This study demonstrates that, unlike in adults, the role of MG/MΦ in injury mechanisms following TBI in the immature brain may not be negative. An improved understanding of MG/MΦ function in paediatric TBI could support translational efforts to design therapeutic interventions.

## Highlights

• TBI in neonates leads to tissue loss and microglia/macrophage activation over several days.
• Microglia/macrophage are predominantly of a reparatory/regenerative or immunomodulatory type after neonatal TBI.
• Microglia/macrophage inhibition (using minocycline) after TBI is only transiently neuroprotective.

## 1 Introduction

Traumatic brain injury (TBI) is the most common injury leading to significant lifelong disability that occurs in children (Stanley et al., 2012). Unfortunately, the cognitive and behavioural deficits caused by traumatic brain injury (TBI) to the immature brain are more severe and persistent than those observed following comparable injuries to the mature (adult) brain (Anderson et al., 2005, Ewing-Cobbs et al., 2006, Hessen et al., 2007, Rivara et al., 2012) (reviewed in (Giza et al., 2007)) with injury in an experimental setting progressing into a chronic brain disorder (Ajao et al., 2012, Kamper et al., 2013). This is in contrast to Kennard’s Principle that the immature brain has superior potential for repair (Bennet et al., 2013). This is of particular concern as children under the age of four years sustain TBI more frequently than any other age group (Koepsell et al., 2011) and in children under the age of 2 years, the rates of TBI serious enough to require intensive care support are as high as 50 per 100,000 (Keenan et al., 2003). A developmental sensitivity to TBI as seen in humans is also observed in a rodent model of TBI, where within the first 30 days of life, injury is maximal when TBI is caused at postnatal day 7 (P7) (Bittigau et al., 1999). In addition, during the first three postnatal weeks, rodents display a heightened sensitivity to excitotoxicity (Ikonomidou et al., 1999). In mouse and humans this period is when developmental processes such as maximal brain growth, synaptogenesis and myelination occur.

In the paediatric population, TBI is caused by injuries and insults, which include acceleration/deceleration injuries (shaken baby syndrome) and contusion injuries (direct skull impact) (Pinto et al., 2012). Contusion injuries are the prevailing form of non-inflicted injuries and also represent a large proportion of inflicted injuries (Pinto et al., 2012). The primary injury process in TBI is mechanical damage (i.e. shear forces inducing vascular damage and bleeding), followed immediately by mast cell degranulation (Stokely and Orr, 2008), and secondary pathological processes, including excitotoxicity, ischemia, mitochondrial dysfunction, activation of matrix metalloproteinases (MMPs) and activation of caspases leading to apoptosis (Xiong et al., 2013). These secondary injury processes induce neuroinflammation, which itself has the potential to be neurotoxic (Hagberg et al., 2012), but which is poorly understood in the immature brain following TBI.

Microglia (MG) are the central regulators of neuroinflammation, involved in the pathological processes of the majority of acute and chronic brain injuries, such as stroke, Alzheimer’s disease and multiple sclerosis (for review see (Prinz et al., 2011)). Thus MG are logical candidates to mediate neuropathological changes following TBI in the immature brain. MG possess enormous functional plasticity that allows them to participate in both injury and repair, as reviewed in (Colton and Wilcock, 2010, Ransohoff and Perry, 2009). The nomenclature of these functional activation states (phenotypes) of MG has been simplified to facilitate their description and a common nomenclature includes classic pro-inflammatory or cytotoxic, anti-inflammatory or reparatory/regenerative and immunomodulatory phenotypes.

There are specific differences in the immune and inflammatory responses to injury between neonatal and adult humans and experimental animals (Copland et al., 2004, Giza et al., 2007, Schultz et al., 2004, Zhu et al., 2005), including in microglia responsiveness (Butovsky et al., 2014). Studies of neuroinflammatory profile and MG activation states have recently been published in adult models of TBI (Bye et al., 2007, Kumar et al., 2015) but it is unknown how microglia would respond to a similar injury to the developing brain. As such, this study investigates for the first time the characteristics of MG- driven neuroinflammation in a mouse model of paediatric TBI. Furthermore, as a proof-of-concept, we aimed to assess the effects of modulating MG activity on injury severity using the immunomodulatory tetracycline minocycline. Minocycline reportedly has strongly anti-inflammatory actions and has been used to reduce MG activation and injury with success in numerous pathological models (see Table 1 and review, (Garrido-Mesa et al., 2013)).

**Table 1:** Summary of selected studies investigating the neurotherapeutic effects of minocycline.

Study	Animal	Injury	Dose	Regime	Cell death / Lesion Volume	MG number	Outcome	
Dommergues et al. (2003)	P5 mouse	Excitotoxic	45 mg/kg	Twice daily from P5-P7	Decreased Cleaved Caspase-3 at +1 day and decreased lesion volume at +5 days	Decreased numbers of Lectin+ MG	Decreased lesion volume at +5 days	
Fox et al. (2005)	P7 rat	MCAO	45 mg/kg	+2 h & +2 h, or +8 h & +18 h	Decreased lesion volume at +1 day	No change in ED1+ MG numbers	No improvement in lesion volume at +7 days	
Yang et al. (2015)	Adult rat	MCAO	5 mg/kg	+5 min	Decreased infarct on MRI	60% decreased (Increased anti-inflammatory type MG)	Improvements on MRI at 4 week	
Cai et al. (2006)	P4 rat	HI	45 mg/kg	12 h before, immediately after & daily for 3 days	Decreased pyknosis at +4 days	50% decrease in numbers of lectin positive MG	Decreased loss of mature oligodendrocytes and myelin at +2 weeks	
Lechpammer et al. (2008)	P6 rat	HI	50 mg/kg	Immediately following HI	Decreased white matter injury at +3 days	Decreased numbers of CD68+ & MHCII+ cells at +3 days		
Arvin et al. (2002)	P7 rat	HI	22.5–45 mg/kg	Immediately before or +3 h	Decreased lesion volume at +7 days	–	Decreased lesion volume at +7 days	
Tsuji et al. (2004)	P7 mouse	HI	22–135 mg/kg	(1) Twice in first 24 h (45 mg/kg) & twice in the next 24 h (23 mg/kg), or (2) Twice in first 24 h (135 mg/kg) & twice in the next 24 h (68 mg/kg), or (3) Single dose 12 h before HI (45 mg/kg)	Exacerbated total injury score for all treatments *(1–3)* at *+*7 days	–	*Mouse:* Exacerbated total injury score for all treatments *(1–3)* at *+*7 days	
	P7 rat		45 mg/kg	(4) Immediately before HI, or(5) 12 h before HI	Decreased total injury score for both treatments *(4–5)* at *+*7 days	–	*Rat:* Decreased total injury score for both treatments *(4–5)* at *+*7 days	
Hanlon et al. (2016)	P11 rat	Repeated TBI (CCI)	45 mg/kg	Once immediately after the third and final TBI	No change in fluro-jade B+ cell number at +3, +7 & +21 days	No change	Exacerbated defects in retention tasks. No improvements in tissue loss or spatial memory defects at +21 days.	
Bye et al. (2007)	Adult mouse	TBI (CCI)	45 mg/kg	+30 min & every 12 h for 3 days	Decreased at +1 day, no change at +4 days	Decreased amoeboid ED1+ MG	No improvement in motor function at +1 week	
Homsi et al. (2010)	Adult mouse	TBI (CCI)	90-45 mg/kg	+5 min (90 mg/kg), +3 h & +9 h (45 mg/kg)	50% decrease in cortical tissue loss	50% Decrease in CD11b+ MG/MΦ	Improvement in locomotor hyperactivity at +8 weeks	
Current study	P7 mouse	TBI (WD)	45 mg/kg	Immediately after & at +24 h & +48 h	Decreased cleaved caspase-3+ cell numbers, decreased ventricular volume at +1 day	15% decrease in numbers of Iba1+ MG (minimal change in activation by gene expression)	No improvements in neuropathology at +5 days	

HI, hypoxic/ischemic. CCI, controlled cortical impact. WD, weight drop.

## 2 Materials and methods

### 2.1 Animals

Study ethics were approved by the Bichat and Robert Debré Hospital ethics committee (No 2011-14/676-0050) and adhered to the European Union Guidelines for the Care and Use of Animals. Procedures were typically carried out between 10am and 1 pm (light phase: 7am-7 pm daily), all animals were monitored daily during experimentation. A single animal represents an experimental unit with groups spread between and across litters where possible and each litter had an approximate 50–50% spread of males-females. Specifically, data in Fig. 2 are derived from 6 litters; Fig. 3 derived from 24 litters; Fig. 4, Fig. 5, Fig. 7 derived from 6 litters each; Fig. 6 derived from 6 litters. Animals were housed in Plexiglas cages (30x18x15 cm) together with littermates and their dam for the whole of the experiment. Animals had access to standard chow and water *ad libitum* and bedding was wood-chips with shredded paper for nesting (Pharmaserv, France).

### 2.2 Traumatic brain injury model and experimental procedure

**Fig. 1:**
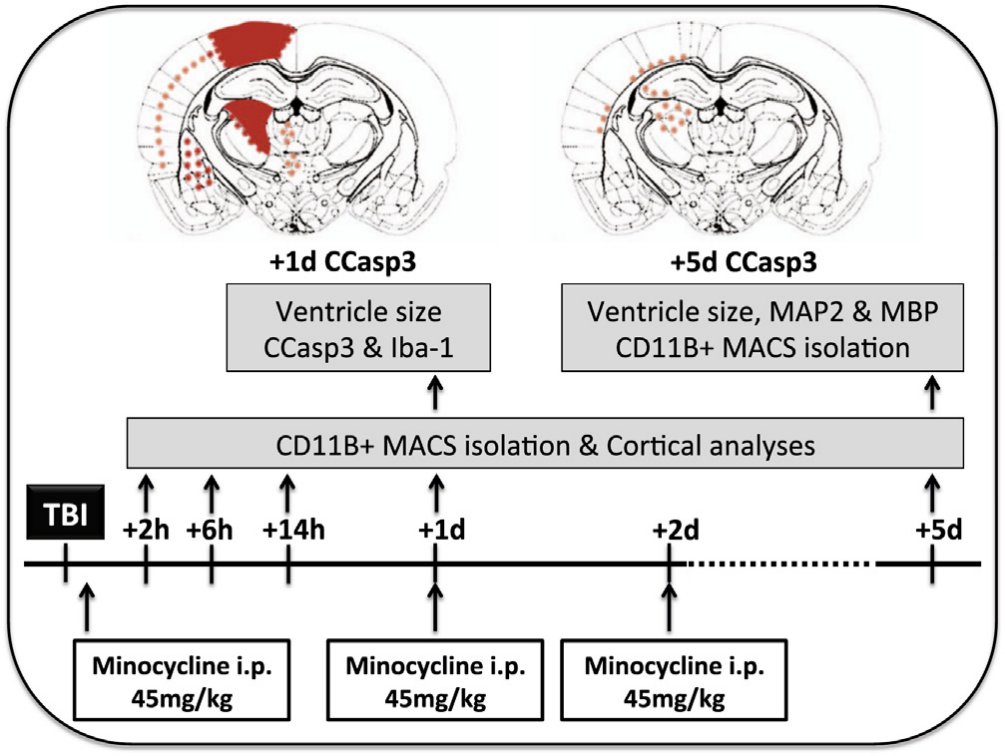
Schematic representation of the experimental procedures including administration of drugs and tissue collection, and injury distribution. Injury is indicated by the expression of CCasp3 at 1 day (+1d) and 5 days (+5d) following TBI. Slightly increased areas of labelling shown by orange stars, moderate increases by red stars and intense changes shown as blocks of red. (For interpretation of the references to colour in this figure legend, the reader is referred to the web version of this article.)

Postnatal day 7 (P7; weight 4–5 g) OF1 mice (Charles River, L’Arbresle, France) of both sexes were randomly (alternating animals) allocated to TBI, control or TBI+ treatment (phosphate buffered saline [PBS] or minocycline) groups. The study protocol is detailed in Fig. 1. A dose of 45 mg/kg of minocycline was chosen based on its prior use in models of adult TBI, stroke and paediatric excitotoxic lesion, see Table 1. In a separate experimental work-space within the animal facility, mice were anesthetized with isoflurane (8% induction) and subjected to a closed head weight–drop head trauma at P7 in a model as described previously (Kaindl et al., 2007). In brief in a process lasting no more than 3 min, the skull was fixed into a stereotaxic frame, the skull surface exposed with a skin incision and the impact device was oriented parallel to the parietal bone with the centre of the foot plate (2 mm diameter) positioned 2 mm anterior and 1 mm lateral to lambda on the parietal bone. The foot-plate was first allowed to touch the skull and was then further depressed by 0.5 mm. The impact device consisted of a hollow stainless-steel cylinder 20 cm in length, perforated at 1 cm intervals to prevent air compression, and guiding a 10 g weight falling from a height of 10 cm onto the foot-plate (2.0 mm in diameter). The contusion impact was delivered unilaterally to the left side of the skull, the same operator conducted all experiments and cortical contusions were of comparable severity in all animals. Body temperature was kept constant via the use of a heating pad maintained at 37 °C until pups were returned to their dams at approximately 15 min post-TBI. Sham animals were anesthetized and an incision made in their scalp, this was then sutured and animals were recovered after 3 min in line with the time taken for the TBI procedure. Minocycline (45 mg/kg in PBS: Sigma, Lyon, France) (Cai et al., 2006, Dommergues et al., 2003) or PBS alone was injected intraperitoneal immediately following TBI, and at 24 and 48 h post-TBI, depending on the protocol. A group of sham minocycline was not included in this study as the specific aim was to investigate the effects of modulating the microglial activation state associated with TBI. Furthermore, minocycline has been widely reported to have no effect on microglial gene expression in a basal state (Kobayashi et al., 2013, Scholz et al., 2015).

### 2.3 Tissue preparation, and histology

One or five days after TBI, animals were euthanatized via an overdose of pentobarbital and decapitation and brains were immersion fixed (formol 4% for 5 days), embedded in paraffin and coronally sectioned (16 μm) from the frontal pole to the occipital lobes. Ventricular area was determined as described previously (Kaindl et al., 2007, Moretti et al., 2016) on cresyl-violet-stained sections. In short, the border of each lateral ventricle from three serial sections spanning the hippocampus and midstriatum was outlined, then the cross-sectional ventricular areas were determined using ImageJ software (version 1.43; National Institute of Health, Bethesda, Md., USA) and the ratio between the left (ipsilateral) and right (contralateral) ventricular areas determined. All tissue processing and analyses were carried out by investigators blind to the treatment group due to coding of the brains and covering of the codes during analysis. There were no differences in the ventricular area of the contralateral hemisphere between sham and TBI mice. Immunohistochemistry (IHC) was performed as previously described (Fleiss et al., 2012), and the antibodies used included: rabbit monoclonal anti-ionized calcium binding adaptor molecule-1 (Iba-1; 1:1000, Wako Chemicals USA, 019-19741), Rabbit monoclonal anti-cleaved caspase 3 (CCasp3; 1:200, Cell Signalling, 9661), mouse monoclonal anti-myelin basic protein (MBP; 1:500, Millipore, MAB382) and mouse monoclonal anti-microtubule-associated protein 2 (MAP2; 1:2000, Sigma, M4403). After overnight incubation with primary antibodies and washing, sections were incubated with appropriate secondary antibodies (1:200; Vectorlabs, California, USA).

### 2.4 Analysis of neuropathology

Ventricular volume was assessed by measuring the area of the ipsilateral and contralateral ventricles and expressed as percentage of change compared to the contralateral values. Iba-1-positive and CCasp3-positive cells were counted in the parietal cortex, hippocampal CA1 region and striatum of the traumatized hemisphere, on two images captured using a Leica DM6000 B microscope (Leica Microsystems Ltd.) and a 10X objective at the level of maximum lesion (approximately −1.50 mm from bregma). Counts were carried out using Image J and cell numbers within a given region expressed as cells/mm^2^. The area of MAP2 and MBP immunolabeling was measured at 4–6 levels per brain (one 16 μm-thick serial section every 576 μm) as previously described (Fleiss et al., 2012). Volumes of MAP2 and MBP immunolabeling were calculated from area measurements according to Cavalieri’s principle using the following formula: V = SA × P × T, where V is total volume, SA is the sum of the areas measured, P is the inverse of the sampling fraction and T is the section thickness. Volume loss was estimated by the difference in calculated volumes between the contralateral (right) and the ipsilateral hemispheres (left).

### 2.5 CD11B-antibody-coupled magnetic cell isolation

At different time-points following TBI (2, 6, 14, 24 h and 5 days), cells positive for CD11B (cluster of differentiation 11 beta, a marker for MΦ and MG), were extracted using the antibody-coupled magnetic bead system (MACS) following the manufacturer’s recommendations (Miltenyi Biotec, Bergisch Gladbach, Germany) and as previously reported (Schang et al., 2014). In brief, the olfactory bulbs and cerebella were removed and the hemispheres mechanically and enzymatically digested using the Neural Tissue Dissociation Kit (Miltenyi Biotec, Germany). Three or four hemispheres were pooled for each sample to ensure sufficient RNA quantities. In a preliminary analysis, comparisons of MG/MΦ activation and cytokine gene expression between left and right sham hemisphere did not show any differences and samples were pooled. Homogenized and digested tissue was incubated with magnetic coupled anti-CD11B antibodies and CD11B-positive cells were separated in a magnetic field before being counted and frozen at −80 °C. The purity of separated cells was assessed using quantitative real-time polymerase chain reaction (qRT-PCR) for glial fibrillary acidic protein (GFAP; astrocytes), MBP (oligodendrocytes), neuronal nuclear antigen (NeuN; neurons) and CD11B (MG/MΦ), and showed levels of contamination less than 5%. We have described the CD11B-positive population extracted from the brain as MG/MΦ as we cannot exclude a contribution of macrophages to the cell population (Hsieh et al., 2013).

### 2.6 RNA extraction and quantification of gene expression by real-time qPCR

MG/MΦ qRT-PCR, primer design, and PCR setups were similar to that previously described (Chhor et al., 2013, Husson et al., 2005, Schang et al., 2014). In brief, RNA was extracted using Qiagen RNA extraction columns as per the manufacturers instructions, including initial homogenisation in Trizol (Invitrogen). RNA purity was verified using a nanodrop. Reverse transcription was performed using an iScript RT kit (Biorad) as per manufacturers instructions. PCR reactions were setup on a loading robot in 384 well plates with Sybr green from Biorad as per recommended protocol. Primer sequences are given in Table 2. *Gapdh* (glyceraldehyde-3-phosphate dehydrogenase) was used to normalize the quantitative experiments based on prior reference-gene suitability testing and we verified for each experiment that the raw *Gapdh* values were not significantly different between groups. The relative quantities are expressed as the specific ratio between the gene of interest and the reference gene. Genes were classified as cytotoxic, repair/regeneration or immunomodulatory based on the literature (Colton and Wilcock, 2010, Ransohoff and Perry, 2009) and previous characterization in our lab (Chhor et al., 2013).

**Table 2:** Primer sequences and NCBI references.

Gene	Sense	Antisense	NCBI Reference
*Gapdh*	GGC CTT CCG TGT TCC TAC	TGT CAT CAT ATC TGG CAG GTT	NM_008084.2
*iNos*	CCC TTC AAT GGT TGG TAC ATG G	ACA TTG ATC TCC GTG ACA GCC	NM_010927.3
*CD32*	CTG GAA GAA GCT GCC AAA AC	CCA ATG CCA AGG GAG ACT AA	NM_010187.2
*CD86*	GAG CGG GAT AGT AAC GCT GA	GGC TCT CAC TGC CTT CAC TC	NM_019388.3
*Ptgs2*	TCA TTC ACC AGA CAG ATT GCT	AAG CGT TTG CGG TAC TCA TT	NM_011198.3
*CD206*	CTT CGG GCC TTT GGA ATA AT	TAG AAG AGC CCT TGG GTT GA	NM_008625.2
*Arg1*	GTG AAG AAC CCA CGG TCT GT	GCC AGA GAT GCT TCC AAC TG	NM_007482.3
*Lgals3*	GAT CAC AAT CAT GGG CAC AG	ATT GAA GCG GGG GTT AAA GT	NM_010705.3
*Igf1*	TGG ATG CTC TTC AGT TCG TG	GCA ACA CTC ATC CAC AAT GC	NM_010512.4
*Sphk1*	TCC AGA AAC CCC TGT GTA GC	CAG CAG TGT GCA GTT GAT GA	NM_001172475.1
*Il1rn*	TTG TGC CAA GTC TGG AGA TG	TTC TCA GAG CGG ATG AAG GT	NM_031167.5
*Il4ra*	GGA TAA GCA GAC CCG AAG C	ACT CTG GAG AGA CTT GGT TGG	NM_001008700.3
*Socs3*	CGT TGA CAG TCT TCC GAC AA	TAT TCT GGG GGC GAG AAG AT	NM_007707.3
*IL1b*	GGG CCT CAA AGG AAA GAA TC	TCT TCT TTG GGT ATT GCT TGG	NM_008361.3
*IL-6*	CAA AGC CAG AGT CCT TCA GA	GCC ACT CCT TCT GTG ACT CC	NM_031168.1
*IL10*	CTC CCC TGT GAA AAT AAG AGC	GCC TTG TAG ACA CCT TGG TC	NM_010548.2
*IL-12a*	TCA CAA CCA TCA GCA GAT CA	TGC AGA GCT TCA TTT TCA CTC	NM_001159424.1
*IL-12b*	ATC CAG CGC AAG AAA GAA AA	AAT AGC GAT CCT GAG CTT GC	NM_008352.2
*IL-18*	TTC GTT GAC AAA AGA CAG CC	TAT CAG TCT GGT CTG GGG TTC	NM_008360.1
*Tnfa*	GCC TCT TCT CAT TCC TGC TT	AGG GTC TGG GCC ATA GAA CT	NM_013693.3
*Cxcl1*	GCA CCC AAA CCG AAG TCA TA	AGG TGC CAT CAG AGC AGT CT	NM_008176.3
*Cxcl10*	GGG TAA AGG GAG GTG GAG AG	GCT TAT TGA AAG CGG TGA GC	NM_021274.2
*Ccl2*	CAT CCA CGT GTT GGC TCA	TCA TTG GGA TCA TCT TGC TG	NM_011333.3
*Ccl3*	TTT TGA AAC CAG CAG CCT TT	CTG CCT CCA AGA CTC TCA GG	NM_011337.2
*Mbp*	CCG GAC CCA AGA TGA AAA C	CTT GGG ATG GAG GTG GTG T	NM_010777.3
*Gfap*	CTC CTG GTA ACT GGC CGA CT	AAG CCA AGC ACG AAG CTA AC	NM_010277.3
*NeuN*	CGA TGC TGT AGG TTG CTG TG	CAG ATA TGC TCA GCC AGC AG	NM_001039168.1
*CD11B*	CTG GTG CTC TTG GCT CTC AT	GGC AGC TTC ATT CAT CAT GT	NM_001082960.1

### 2.7 Protein extraction procedure and multiplex cytokine/chemokine assay

Frozen cortices from 6, 14 and 24 h post-TBI were homogenized in 0.1 M PBS, and extracts sonicated in ice-cold homogenization buffer (3 mM ethylenediaminetetraacetic acid [EDTA] and 1% protease inhibitor cocktail, [P8340, Sigma] in 0.1 M PBS) and centrifuged (800xg for 10 min). The supernatant was collected and stored at −80 °C. Protein concentrations were determined via a bicinchoninic acid (BCA) assay. After thawing on ice, supernatants were centrifuged briefly to remove particulates (300*g* for 10 min). Cytokine and chemokine levels were measured using a 96-well magnetic plate assay on a Bio-Plex 200 according to the manufacturer’s instructions (BioRad laboratories, Marnes la Coquette, France). Cytokines and chemokines measured included interleukins (IL) IL-1α, IL-1β, IL-3, IL-4, IL-5, IL-6, IL-9, IL-10, IL-12(p40), IL-12(p70), IL-13, IL-17, granulocyte colony stimulating factor (G-CSF), interferon (IFN) γ, tumor necrosis factor (TNF) α, chemokine C-X-C motif ligand (CXCL) 1 (KC), chemokine ligand (CCL) 2 (also known as MCP-1), CCL3 (also known as MIP1a), CCL4 (also known as MIP1b) and CCL5 (also known as RANTES). All samples were run in duplicate and data analysed with Bio-Plex Manager 6.0 software. Cytokines and chemokines were classified as cytotoxic, repair/regeneration or immunomodulatory based on the literature (Colton and Wilcock, 2010, Ransohoff and Perry, 2009) and previous characterization in our lab (Chhor et al., 2013).

### 2.8 Statistics

Data are presented as means ± SEM. No animals were excluded from any analysis. Numbers in each experiment are indicated within the text, or in the figure legends. Sample sizes were based on calculations of effect sizes from previous studies on this model within the laboratory (Kaindl et al., 2007). For two experimental groups, t-tests or Mann Whitney *U* test were performed. Where more than two experimental groups were compared an ANOVA was performed and when this was significant (p < 0.05) a *Bonferroni* post-test was performed. The appropriate statistical test was chosen based on data normality (Kolmogorov-Smirnov test). The statistical test performed on each data set (using GraphPad 5.0 software [San Diego, CA, USA]) is indicated in the figure legend or within the text.

## 3 Results

### 3.1 TBI increases early cell death, microglial number and cortical cytokine / chemokine levels

**Fig. 2:**
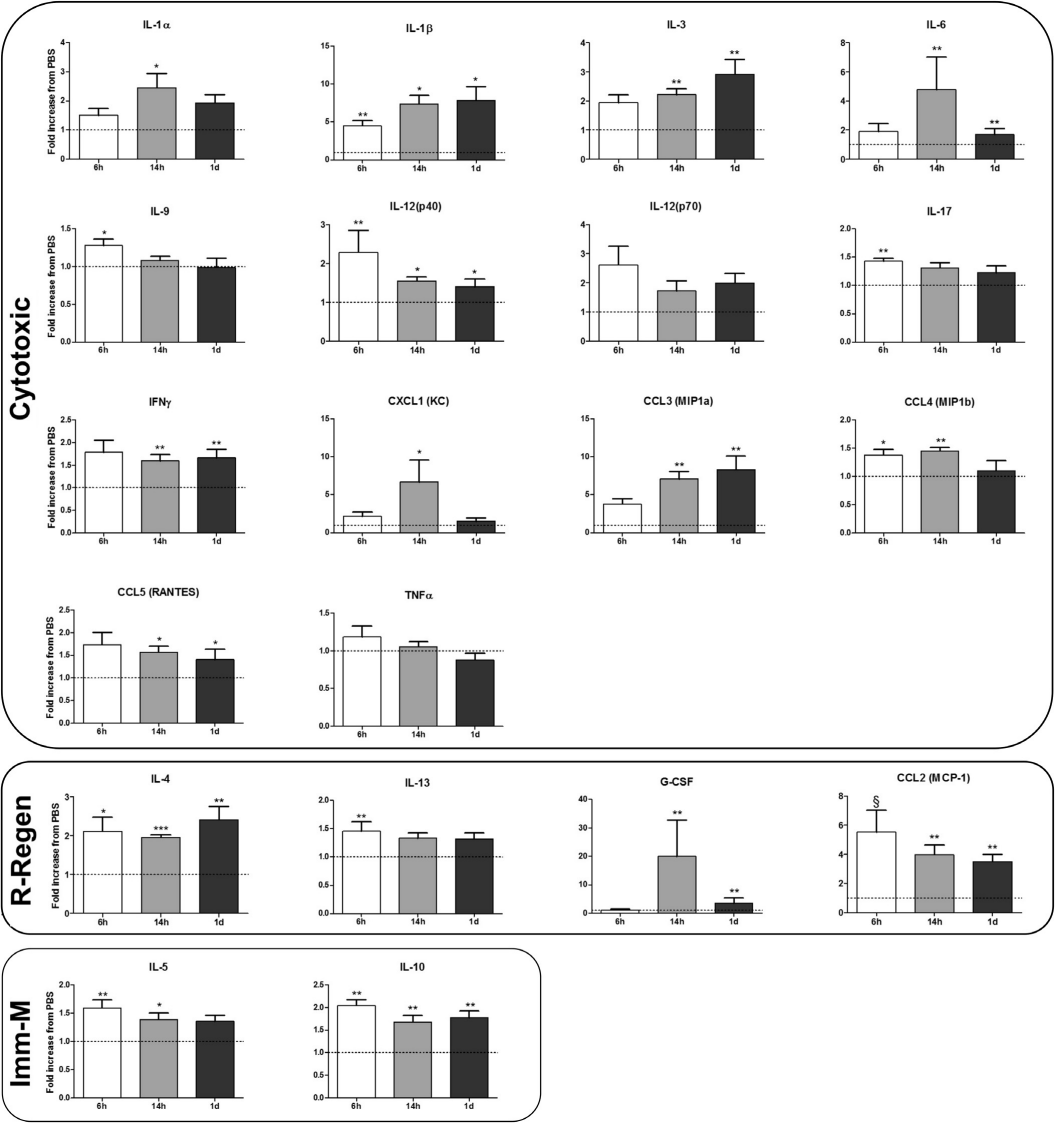
Expression of cytokines and chemokines from the ipsilateral hemisphere over time post-TBI. Genes are grouped based on predicted role in inflammation: cytotoxic (CytoT), reparatory/regenerative (R-Regen), and immunomodulatory (Immu-M) based on (Colton, 2009, Prinz et al., 2011). Data are normalized to sham group expression (Sham = 1) and are indicated as means ± SEM (n = 6–8 animals/group). Data were compared to the corresponding sham group using a Mann-Whitney *U* test. ^*^p < 0.05, ^**^p < 0.01, ^***^p < 0.001.

Following TBI, CCasp3-positive cells were present in the underlying cortex, thalamic nuclei, hippocampal dentate gyrus, subiculum and striatum, and increased numbers of Iba-1 positive cells were observed mainly in the underlying cortex, hippocampus and striatum, and a qualitative representation is found in Fig. 1. Specifically for Iba-1, numbers of positive cells were increased in the cortex following TBI at P1 by ≈150% (contralateral 30.86 ± 2.24 versus ipsilateral 46.64 ± 2.41; n = 36) and in the striatum by ≈350% (contralateral 12.05 ± 0.77 versus ipsilateral 47.23 ± 2.36; n = 36). We analysed the number of Iba-1 positive cells by sex and found no difference between control values for the cortex (contralateral male 25.21 ± 0.74, n = 20 versus contralateral female 23.97 ± 1.17, n = 16; p = 0.35, *t*-test) or striatum (contralateral male 11.68 ± 1.07, n = 17 versus contralateral female 12.47 ± 1.15, n = 16; p = 0.55, *t*-test), or in the response to injury in the cortex (ipsilateral male 53.71 ± 2.31, n = 17 versus ipsilateral female 51.66 ± 2.48, n = 16; p = 0.52, *t*-test) and striatum (ipsilateral male 47.97 ± 3.38, n = 17 versus ipsilateral female 46.43 ± 3.37, n = 16; p = 0.76, *t*-test). As such, we grouped males and females for the following analysis. The effects of TBI on the expression of 20 cytokines and chemokines were measured in the ipsilateral hemisphere at 6, 14 h and 24 h, and compared to levels in sham animals (Fig. 2). Expression increased for markers associated with each MG/MΦ phenotype at all three time points. We observed that relative to levels in a sham hemisphere, the pro-inflammatory/cytotoxic phenotype markers IL-1β and CCL3 (MIP1α) showed the greatest and most persistent increases in expression over time (>5 fold). The prototypical anti-inflammatory or reparatory/regenerative cytokine IL-4 and the immunomodulatory cytokine IL-10 were also increased at all three time points in the ipsilateral hemisphere. TNFα and IL-12 (p70) were the only markers that did not significantly increase at any time point.

### 3.2 TBI induces MG/MΦ expression of markers of a regenerative/immunomodulatory phenotype

**Fig. 3:**
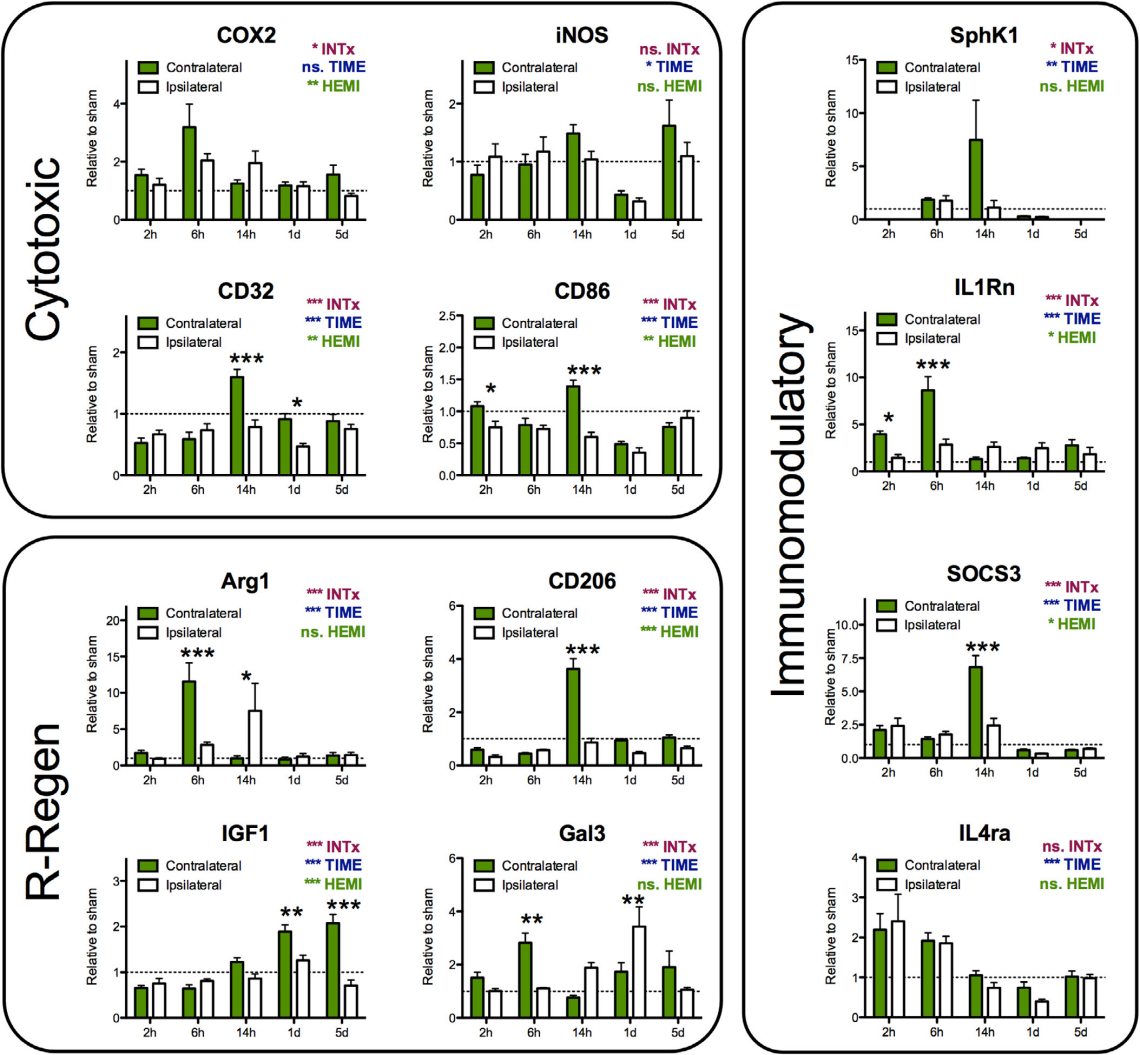

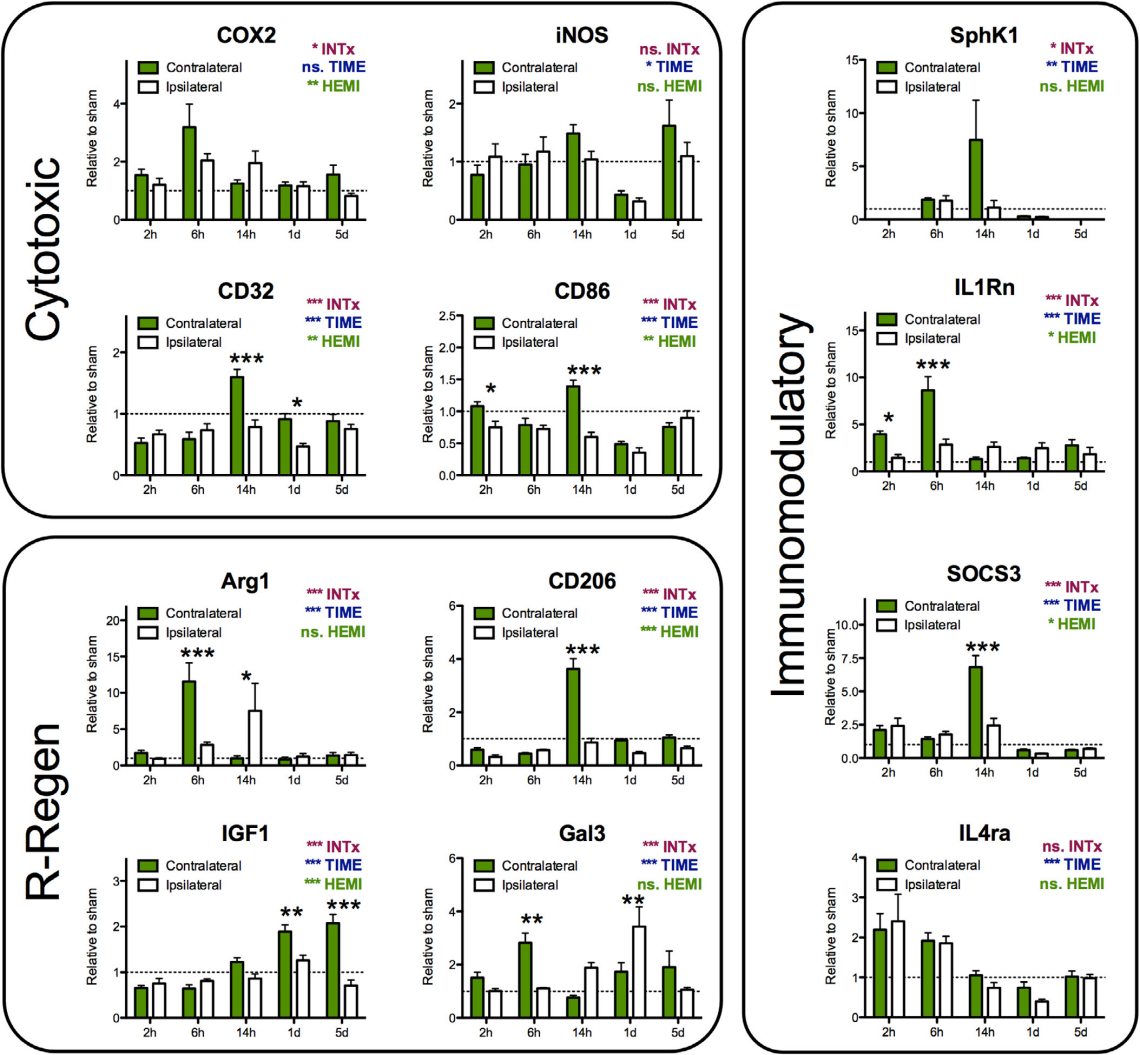
Expression of phenotype markers by MG/MΦ isolated at various times post-TBI. Genes are grouped based on predicted role in inflammation: cytotoxic, reparatory/regenerative (R-Regen), and immunomodulatory based on (Colton, 2009, Prinz et al., 2011). Data are normalized to sham group expression (Sham = 1) and are means ± SEM (n = 5–6 animals/group). Gene expression over time was analysed with a two way repeated measures ANOVA, with a Bonferroni post-test to compare the relative expression for each hemisphere at each time point. Summary of the ANOVA results are presented on each panel (effects of interaction between variable [INTx], effects of time [TIME] and effects of TBI [HEMI]. Results of the post-test are indicated with: ^*^p < 0.05, ^**^p < 0.01, ^***^p < 0.001.

CD11B-positive MG/MΦ were isolated from whole cortices using MACS technology at 2, 6 and 14 h and 1 and 5 days post-TBI, and gene expression of 12 phenotype markers was measured (Fig. 3). Expression of the prototypical cytotoxic MG/MΦ markers CD86 and CD32 was decreased by TBI, and iNOS showed no increase at any time point examined. The cross-phenotype marker Cox-2 (cytotoxic-immunomodulatory) and IL1Rn, which has immunomodulatory functions, were persistently increased by TBI. Two additional immunomodulatory markers, SOCS3 and IL-4rα, showed early increases, but by 1-day post-TBI were reduced to below non-TBI levels. Among the reparatory/regenerative MG/MΦ markers, Arg1 and Gal3 showed persistent increases following TBI, but IGF-1 and CD206 were decreased.

### 3.3 Blocking MG/MΦ activation with minocycline causes improvements in neuropathology at 1 day post-TBI

**Fig. 4:**
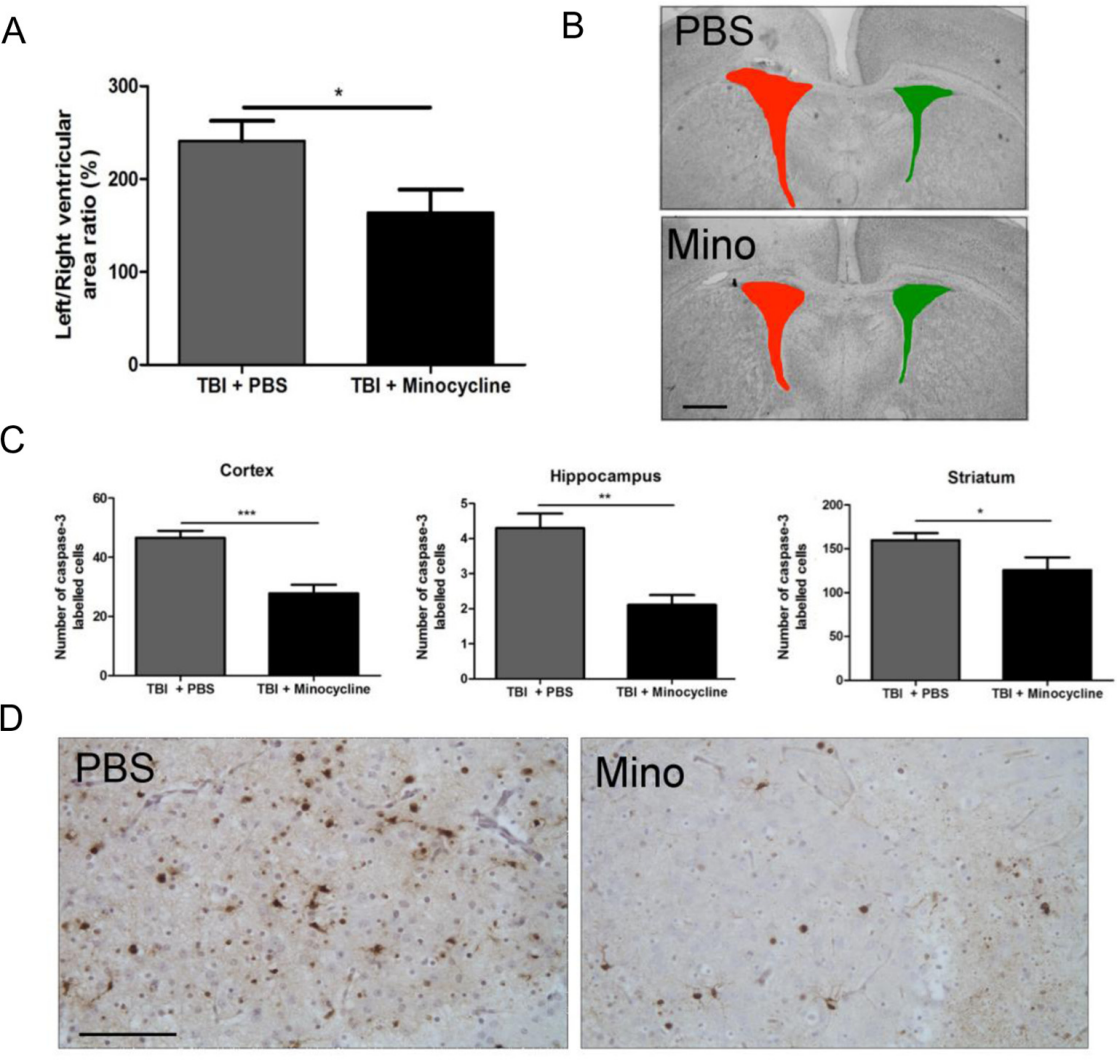
Minocycline improves neuropathology 1-day post-TBI. A) Quantification of ventricular volume at 1 day post-TBI, and B) representative images of cresyl-violet-stained sections from both groups indicating ventricular size. C) Quantification of CCasp-3-positive cell number. D) Representative images of CCasp-3 immunolabeling in the striatum from both groups, scale bar 50 μm. Data are indicated by means ± SEM (n = 9–18 animals/group), and PBS- and minocycline-treated groups were compared via a Student’s *t*-test. ^*^p < 0.05, ^**^p < 0.01.

Early brain injury was assessed via CCasp3 cell counts and using ventricular dilatation, calculated as the ratio of the ventricular size in the ipsilateral vs. the contralateral hemisphere. Animals treated with minocycline had reduced numbers of CCasp3-positive cells in the cortex, hippocampus and striatum (Fig. 4C and D). In agreement with these data, minocycline treatment led to less ventricular dilatation compared with the untreated group at 1 day post-TBI (Fig. 4A and B). Sham groups treated with either vehicle or minocycline displayed no change in ventricular size and had very low levels of CCasp3-positive cells (data not shown).

### 3.4 Improved neuropathology due to minocycline is accompanied by reduced MG number and altered MG/MΦ activation

**Fig. 5:**
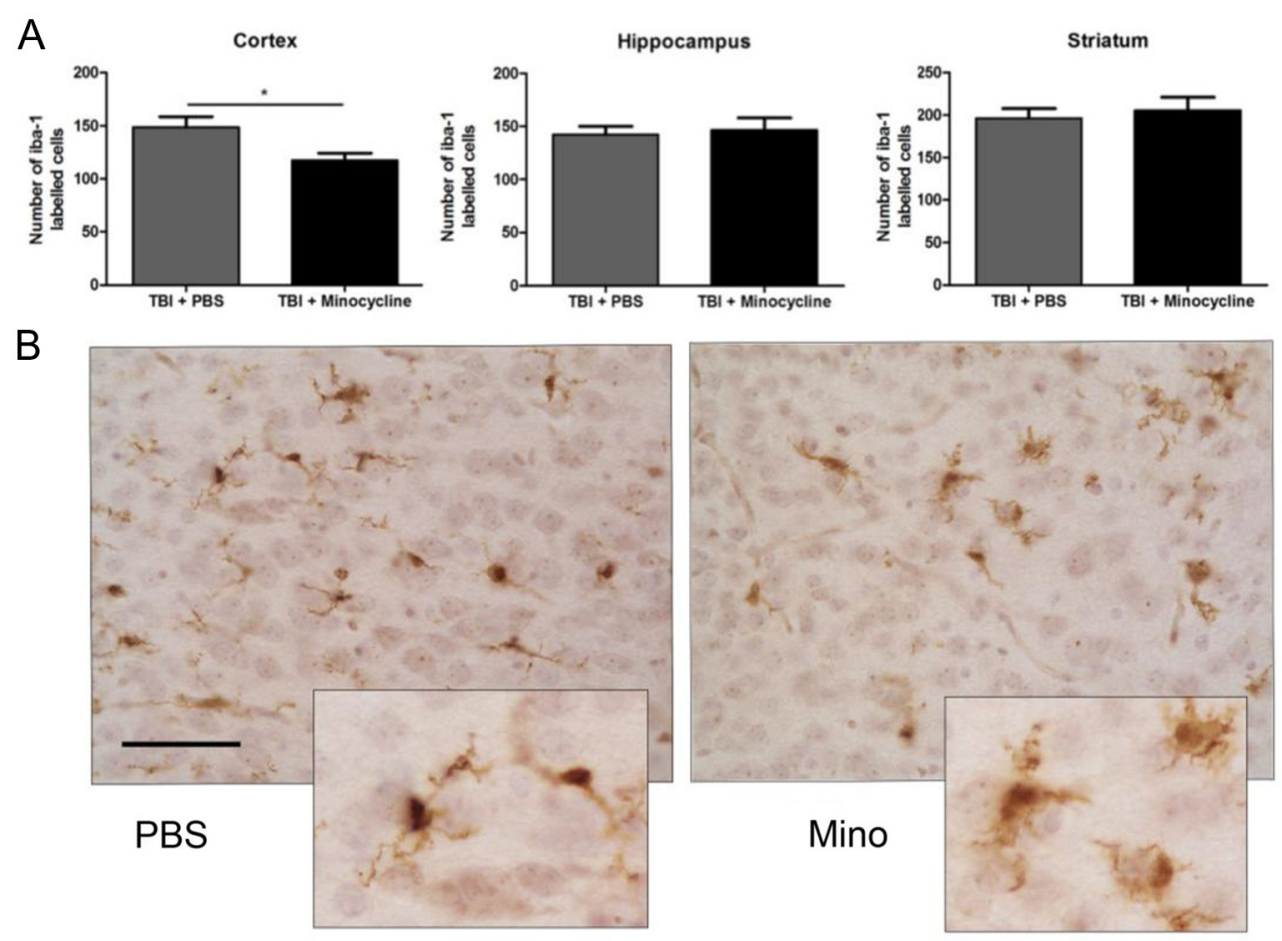
Minocycline decreases MG cell number 1-day post-TBI. A) quantification of the number of Iba-1-positive cells at 1 day following TBI in PBS- and minocycline-treated mice within the cortex, hippocampus and striatum. B) Representative Iba-1-positive cells in animals from each group from within the striatum, scale bar 50 μm. Data are indicated by means ± SEM (n = 9–16 animals/group). PBS- and minocycline-treated groups were compared via a Student’s *t*-test. ^*^p < 0.05.

**Fig. 6:**
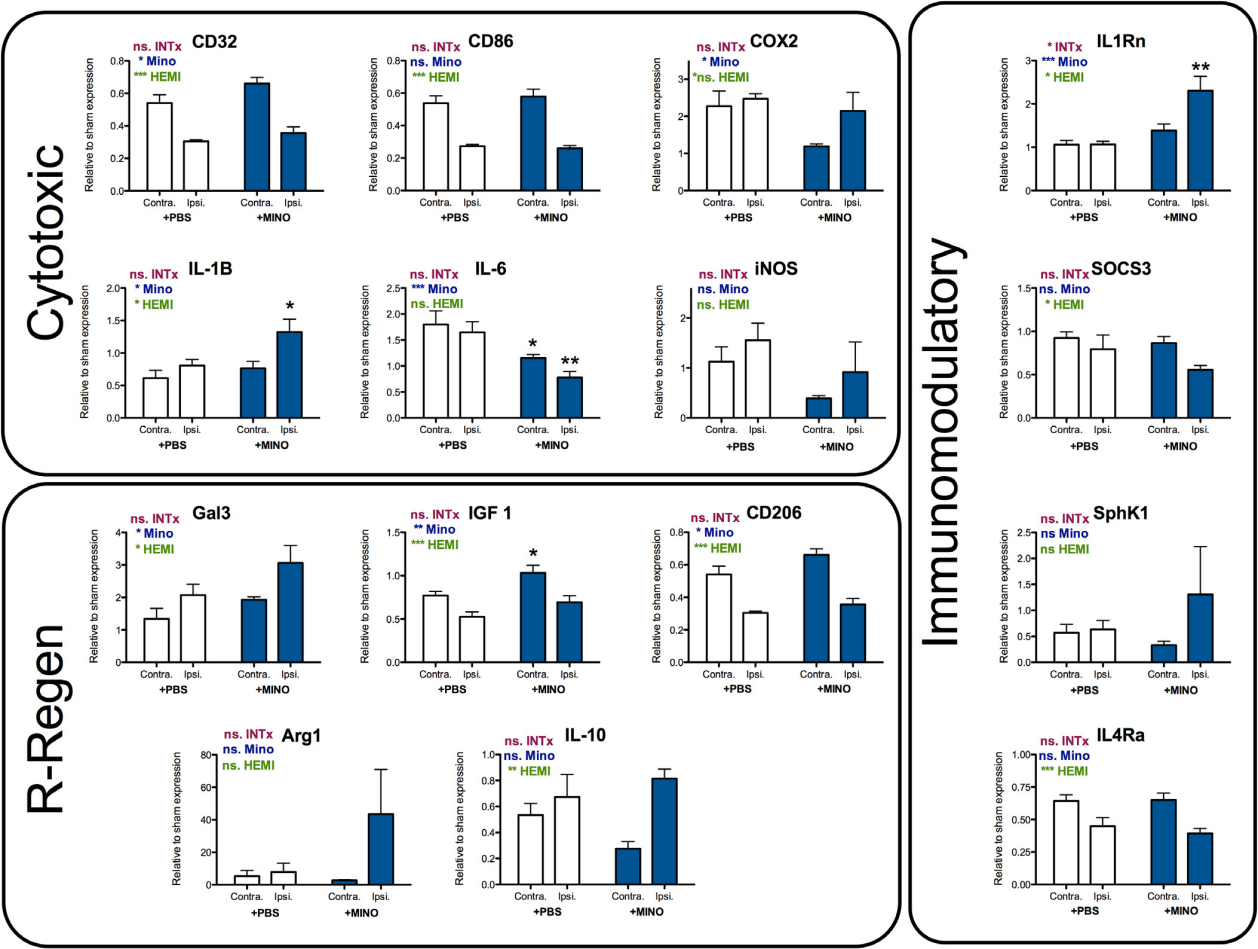
Effects of minocycline treatment on the expression of phenotype markers from MG/MΦ isolated 24 h post-TBI. Data are shown normalized to expression in a sham group (Sham = 1) and as means ± SEM (n = 9–16 animals/group). Genes are grouped based on predicted role in inflammation: cytotoxic, reparatory/regenerative (R-Regen), and immunomodulatory based on (Colton, 2009, Prinz et al., 2011). Data were compared with a two way ANOVA, with a Bonferroni post-test to compare the relative expression for each hemisphere (PBS versus TBI). Summary of the ANOVA results are presented on each panel (effects of interaction between variable [INTx], effects of time [TIME] and effects of TBI [HEMI]. Results of the post-test comparing each hemisphere are indicated with: ^*^p < 0.05, ^**^p < 0.01, ^***^p < 0.001.

To characterize any relationship between the MG/MΦ inflammatory response and neuroprotection, 1 day following TBI, MG cell numbers were quantified using Iba-1 immunolabeling and the phenotype of isolated CD11B-positive MG/MΦ was assessed. As expected based on previous reports of the effects of minocycline, the numbers of Iba-1-positive cells were decreased in the cortex, hippocampus and striatum of minocycline-treated animals (Fig. 5). Minocycline induced complex changes in the phenotype and cytokine/chemokine expression of MG/MΦ that were isolated post-TBI (Fig. 6). The cytotoxic phenotype markers iNOS and IL-6 were reduced from TBI only levels by minocycline treatment but IL-1β was increased. Among repair/regeneration markers, treatment with minocycline stimulated a further increase in the expression of Gal3 and Arg1, and reduced the typical loss of IGF1. Among immunomodulatory markers, minocycline increased the expression of IL-1Rn above typical levels but led to an even greater decrease in the expression of IL10.

### 3.5 Early improvements in neuropathology due to minocycline treatment are lost by 5 days post-TBI

**Fig. 7:**
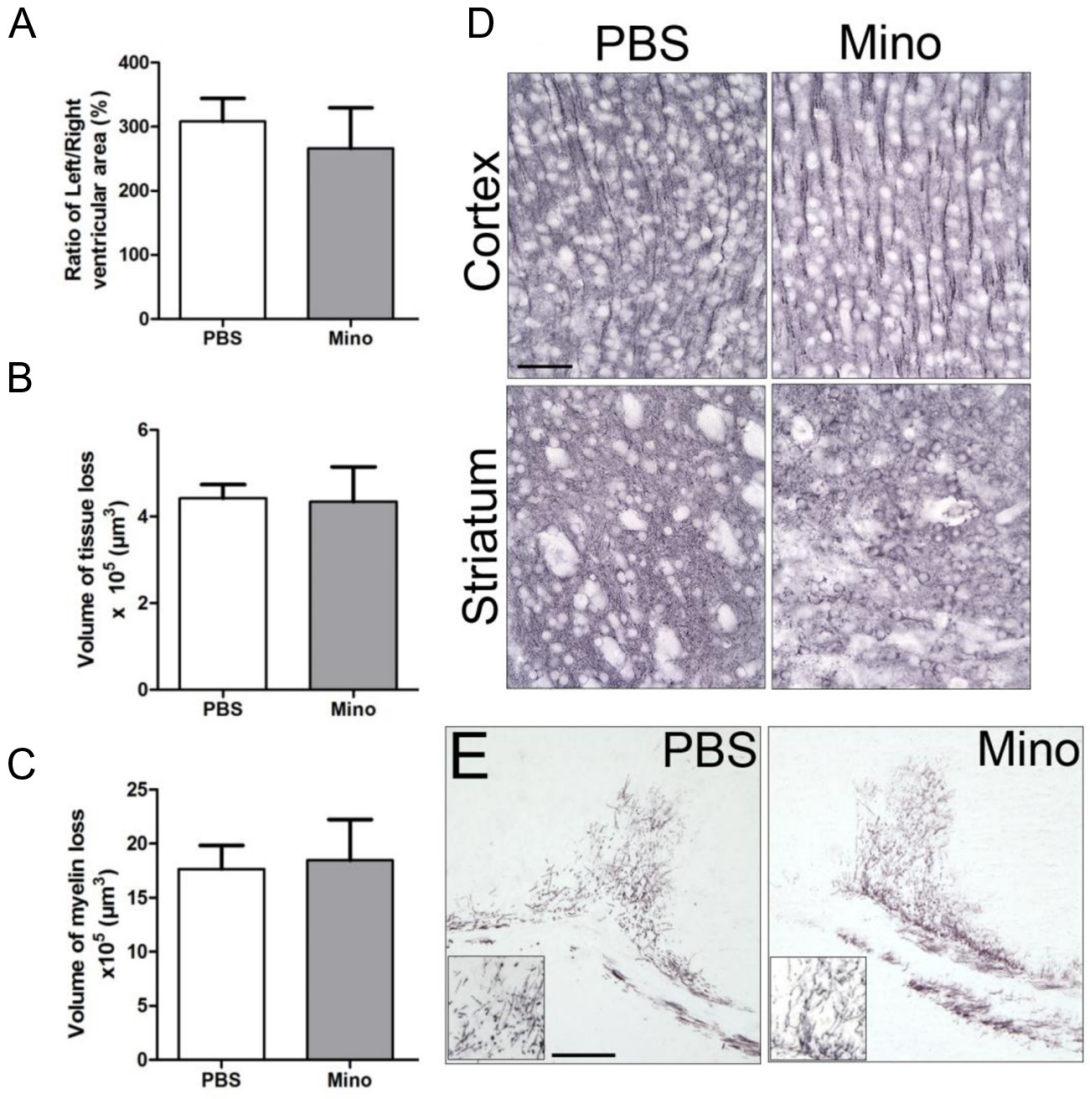
Lack of improvement in neuropathology in minocycline treated animals 5 days post-TBI. A) Quantification of ventricular size 5 days post-TBI and B) quantification of the volume of tissue loss in the traumatized hemisphere 5 days post-TBI based on MAP-2 immunoreactivity, D) representative photomicrographs of MAP2-immunolabeled sections, scale bar 50 μm. C) and E) quantification and representative images of the volume of white matter loss in the traumatized hemisphere based on MBP immunoreactivity, scale bar 200 μm. Data are indicated by means ± SEM (n = 12–18 animals/group). PBS- and minocycline-treated groups were compared via a Student’s *t*-test.

Immunolabeling for MAP2 and MBP were used as surrogates for damage to neurons and myelination by oligodendrocytes respectively. TBI decreased the volume of tissue immunolabeled for MAP2 and MBP, and persistently caused ventricular dilatation in the injured hemisphere at 5 days post-TBI (P14; Fig. 7). MAP-2 immunolabeling was similar to that reported previously in the immature brain (Carlsson et al., 2011, Lingwood et al., 2008), displaying a more diffuse pattern and with less cytoplasmic intensity than in the adult. Despite improvements at 1-day post-TBI, at 5 days post-TBI, in animals treated with minocycline, ventricle size was identical to that in PBS treated TBI animals (Fig. 7A). In accordance with the ventricular data, the loss of MAP2 and MBP immunolabeling was not prevented by treatment with minocycline (Fig. 7B–E).

## 4 Discussion and conclusions

### 4.1 Principal results and the TBI model

In our closed-contusion model of paediatric TBI, injury modestly increased the levels of both pro- and anti-inflammatory cytokines/chemokines in the brain as well as the number of MG. Isolated MG/MΦ had only moderate changes in gene expression, and increases specifically in markers for the repair/regeneration and immunomodulatory phenotypes. Blocking inflammation/MG/MΦ activation with minocycline decreased MG number, reduced expression of some pro-inflammatory cytokines but was only transiently neuroprotective.

We chose for this study a closed-contusion weight-drop TBI model as it has injury mechanisms similar to those seen in paediatric TBI (Xiong et al., 2013). In particular, within the first 30 days of life, at 7-day old, mice display the most widespread apoptotic injury following TBI (Bittigau et al., 1999). This is also the period of greatest vulnerability to excitotoxic lesion in the rodent (Ikonomidou et al., 1989, McDonald et al., 1988) a likely effect of the reduced compensatory anti-oxidant defences of the immature as opposed to the adult brain (Fan et al., 2003). As apoptotic cell death, excitotoxicity and oxidative stress play crucial roles in the pathogenesis of TBI in the neonate (Ruppel et al., 2001, Zhang et al., 2005); this adds weight to the relative usefulness of modelling TBI at this period of rodent development.

### 4.2 Cortical tissue damage and injury response following paediatric TBI

Following TBI we observed increased total cortical expression of cytokines and chemokines, as well as dilated ventricles and obvious tissue injury in the thalamus and hippocampus of the injured hemisphere. These observations are generally in agreement with previous reports from this model and TBI in large animal models and humans (Helmy et al., 2011, Kaindl et al., 2007, Moretti et al., 2016, Xiong et al., 2013). Specific comparisons across studies are hampered by differences in models and methods, specifically the use of protein versus gene analysis. However, specific comparisons for gene expression can be made to published gene array data. In this context levels of almost all of the jointly reported cytokines and chemokines are considerably lower in our model than that previously reported in two adult rat models of controlled cortical impact (Matzilevich et al., 2002, Raghavendra Rao et al., 2003, White et al., 2013), and one rat model of fluid-percussion injury (Truettner et al., 2005), but were comparable to that reported in a model of adult rat bilateral prefrontal cortical contusion (He et al., 2004). Protein levels for chemokines and cytokines are also consistently and persistently high in previous reports from adult models of contusion and blast-induced TBI (Bye et al., 2007, He et al., 2004, Kumar et al., 2015, Williams et al., 2007). We suggest that further work is needed to ascertain if any smaller magnitude of cortical pro-inflammatory cytokine release is linked to our observation that neonatal TBI induces a predominantly reparatory/regenerative or immunomodulatory MG/MΦ phenotype. As MG/MΦ are the chief drivers of neuroinflammation, a predominantly anti-inflammatory response might prevent cortical inflammation reaching the levels seen in adult injury models, in which there is a robust cytotoxic/pro-inflammatory MG profile (Kumar et al., 2015).

We also wish to briefly discuss the effects of TBI in the contralateral hemisphere. We noted that gene expression was lower than sham level even in the contralateral hemisphere in MG/MΦ for cytotoxic markers (including CD86 and CD32) and also for the repair-regeneration marker CD206 and the immunomodulatory marker SphK1. This type of remote gene expression change has been previously reported in an adult cortical contusion model where it was reported that these are not simply reduced magnitude changes spilling over from the ipsilateral cortex but that some effects are specific (White et al., 2013). Remote tissue changes (such as in the cortex following spinal cord injury) are considered to be crucial mediators of sensorimotor dysfunction and cognitive impairments (Ajao et al., 2012, Kamper et al., 2013, Kim et al., 2006). The diffusion of inflammatory products setting up a chain reaction, signalling via gap junctions in astrocytes and changes in neuronal activity patterns in distant areas are hypothesized to underpin these remote effects. Disruption of the developmental functions of MG (such as synaptogenesis) is considered to underpin some of the injury associated with damage to the immature brain at the injury site and in the remote regions, see (Tremblay et al., 2011). Furthermore, on going changes in homeostatic functions are associated with neurodegeneration such as in aging (Grabert et al., 2016, Griffin et al., 2006, Hart et al., 2012, Lourbopoulos et al., 2015). As such, additional longitudinal studies in this model are warranted to explore the remote and persisting effects of TBI.

### 4.3 MG/MΦ phenotype in paediatric vs. adult TBI and other injury models

This is the first study to assess the phenotype of ex-vivo MG/MΦ over such a comprehensive time course (and with so many markers) after an acute neonatal injury. However, there are interesting temporal studies on isolated MG in an adult TBI model (Kumar et al., 2015), and total cortical inflammation in an adult TBI model (Wang et al., 2013) although the methods of analysis vary making it difficult to directly compare data. However, in adult TBI, the protein expression of *ex vivo* MG for classical pro-inflammatory markers increased over time such that at +5 days all markers were robustly increased, compared to our gene expression data wherein only three cytotoxic markers were moderately increased, with no cohesive time point of change. The authors of the adult TBI study sought to describe the robust predominantly pro-inflammatory or mixed phenotype that replaced a transient but specific repair/regeneration and immunomodulatory phenotype as “Mtran” (Kumar et al., 2015). In our paediatric model, since cytotoxic MG/MΦ gene expression was generally low, including at 5 days post-TBI, we conclude it is unlikely that this phenotype occurs in this model, although we would need to validate our data with the same FACS based protein analysis. However, when comparing neonatal and adult studies it is worth considering that robust age-dependent differences in MG/MΦ gene expression have been reported (Bennett et al., 2016, Butovsky et al., 2014). In brief, the stage of development is likely important for MG/MΦ reactivity and is an important consideration for studies of neuroinflammation.

Another key point in the interpretation of these data is the ability of cells to co-express markers, reflecting the *in vivo* complexity of phenotype descriptors. Our gene expression analysis dose not allow up to determine if there are discrete populations of cells switching phenotype or cell co-expressing different category marker as has been previously reported in adult studies (Bedi et al., 2013, Li et al., 2014, Vogel et al., 2013). Co-expression pattern are likely in our paediatric TBI model, but from the paucity of gene expression changes overall any robust co-expression of markers seems unlikely.

### 4.4 Mode of action of minocycline as a neuroprotective agent

Minocycline is a second-generation semi-synthetic tetracycline that is best known for reducing pro-inflammatory responses via its effects on MG/MΦ (Homsi et al., 2010, Kobayashi et al., 2013, Ng et al., 2012). Minocycline has been used successfully to reduce brain damage across a diverse range of injury/disease models, such as multiple sclerosis (experimental autoimmune encephalitis), term and preterm brain injury (excitotoxicity and hypoxia-ischemia, respectively) and Alzheimer’s disease, for review see (Garrido-Mesa et al., 2013). Minocycline successfully reduced TBI severity at our early time point of 1 day post-lesion, despite there being little pro-inflammatory response from MG/MΦ. It is not clear from the literature whether minocycline can act directly on astrocytes (Kernt et al., 2010, Yoon et al., 2012) to facilitate any effect. Nevertheless, several pathological mechanisms involved in TBI are counteracted by minocycline, possibly accounting for the neuroprotection. These include that minocycline increases levels of the anti-apoptotic protein Bcl-2 (Wang et al., 2004) and the chelation of magnesium and calcium (Gonzalez et al., 2007) and also decreases activation of MMPs (Koistinaho et al., 2005) and caspase-1 and caspase-3 (Sanchez Mejia et al., 2001). The protective effect of inhibiting MMPs in this model of paediatric TBI has been previously demonstrated (Sifringer et al., 2007).

### 4.5 Reasons for absence of long-term neuroprotection by minocycline

This study is not the first to report a limited neuroprotective effect of minocycline (Fernandez-Gomez et al., 2005, Fox et al., 2005, Sriram et al., 2006, Yang et al., 2003). Of particular interest is a transient neuroprotective effect reported in an adult closed-contusion TBI model that is strikingly similar to what we observed. In this adult TBI model, behavioural improvements and reduced lesion volume at 1-day post-TBI were lost by 4 days post-TBI (Bye et al., 2007). An early but transient therapeutic effect of minocycline has also been reported following hypoxic-ischemic injury in the mouse (Fox et al., 2005, Nijboer et al., 2010), indicating that this effect of minocycline is not specific to TBI. A limitation of these previous studies and our current study is that we did not test whether behavioural outcomes were improved, despite no change in neuropathology. The concept of a protective phase of the MG/MΦ response after injury has gained enormous support from studies of adult and neonatal models (Faustino et al., 2011, Hernandez-Ontiveros et al., 2013, Hu et al., 2012, Lalancette-Hebert et al., 2007). We speculate that the lack of persistent neuroprotection with minocycline in this model might be because microglia are attempting to repair the brain. As such, there are short-term positive effects (that might relate to positive effects of minocycline on other cells Koistinaho et al., 2005), but when MG are prevented from attempting to repair the brain in the longer term due to exposure to minocycline these positive gains are neutralised by 5 days. What is apparent however is that as outlined in a (non exhaustive) list of studies in Table 1 there are unclear influences on outcome of species (rat versus mouse) and treatment regime (immediate/early only, versus immediate and continuing). The multitude of differences in experimental conditions and outcome measures preclude any firm conclusions on the influences of these factors on the true neuroprotective ability of minocycline.

## 5 Conclusions

In summary, despite cortical inflammation and cell death following TBI, MG/MΦ retain the expression of markers of an endogenous repair and regenerative phenotype in this model. Also, there are only moderate increases in total cortical inflammatory markers compared to adult injury models. We identified that using minocycline to modify the activity of MG/MΦ had positive early effects on injury, but did not persistently improve outcome. This work adds considerably to our understanding of neuroinflammation after TBI in a neonatal model by suggesting that further therapy design should focus on supporting repair and regeneration type MG/MΦ activation states rather than blanket immunosuppression.

## Conflict of Interest Statement

All authors declare that there are no conflicts of interest.
